# Structure-Guided Covalent Design and Biological Evaluation of Glycidamido-Leucinamide Antivirulence Agents Against *Pseudomonas aeruginosa*

**DOI:** 10.3390/ijms27198465

**Published:** 2026-09-23

**Authors:** Xiaoqing Mu, Yujing Huang, Haoming Chen, Xiaojie Huang, Congzhen Shen, Shengrong Li, Junxia Zheng

**Affiliations:** 1 College of Pharmacy, Shihezi University, Shihezi 832003, China; 2 College of Pharmacy, Jinan University, Guangzhou 510632, China; 3 School of Biomedical and Pharmaceutical Sciences, Guangdong University of Technology, Guangzhou 510006, China

**Keywords:** *Pseudomonas aeruginosa*, antivirulence, quorum sensing, LasB, covalent inhibitor, molecular modelling

## Abstract

Antimicrobial resistance in *Pseudomonas aeruginosa* has intensified the need for therapies that do not rely solely on bactericidal activity. Antivirulence agents attenuate pathogenic functions and may complement conventional antibiotics. The elastase LasB is a major quorum-sensing (QS)-controlled virulence factor of *P. aeruginosa*. Starting from the validated *N*-aryl/α-isobutyl recognition motif of LasB inhibitors, we used an integrated computational workflow combining chemical-space mapping of known LasB inhibitors, focused covalent-library enumeration, and Glu141-directed covalent docking to design glycidamide analogues bearing an epoxide-containing motif. Two compounds were synthesised and characterised, **QSI-1** (4-(trifluoromethyl)phenyl) and **QSI-2** (4-fluorophenyl), forming a matched molecular pair that differs only at the para substituent. Both compounds inhibited biofilm formation at sub-micromolar concentrations (**QSI-1**, IC_50_ = 0.28 μM; **QSI-2**, IC_50_ = 0.51 μM) and directly inhibited purified recombinant LasB, with IC_50_ values of 2.98 μM and 10.20 μM, respectively. Both also preferentially reduced lasB promoter activity relative to the rhlA and pqsA reporters. **QSI-1**, selected for further evaluation, reduced virulence-factor production and surface-associated motility without measurably inhibiting bacterial growth. **QSI-1** enhanced the activity of azithromycin in vitro, and the combination of **QSI-1** (25 μM) with azithromycin (0.05 μg/mL) accelerated wound closure and reduced bacterial burden in a murine excisional wound-infection model. **QSI-1** showed low cytotoxicity toward mammalian cells at concentrations up to 200 μM. These findings identify **QSI-1** and **QSI-2** as covalently designed LasB-active antivirulence leads, while direct experimental confirmation of Glu141 covalent modification remains to be established.

## 1. Introduction

The global rise in antimicrobial resistance (AMR) is among the foremost threats to human health. The most recent Global Burden of Disease analysis estimated that 1.14 million deaths were directly attributable to bacterial AMR, and it was associated with 4.71 million deaths in 2021, with these figures projected to increase to 1.91 million attributable deaths and 8.22 million associated deaths annually by 2050 [1]. *Pseudomonas aeruginosa*, a Gram-negative opportunistic pathogen, represents an important component of this global challenge. In the World Health Organization’s 2024 Bacterial Priority Pathogens List, carbapenem-resistant *P. aeruginosa* is classified as a high-priority pathogen for research, development, and public-health action [2]. Its clinical persistence is facilitated by intrinsic and acquired resistance mechanisms together with substantial genetic adaptability [3]. *P. aeruginosa* is a major cause of hospital-acquired infection in burns and chronic, surgical, and diabetic wounds, as well as chronic lung infection in cystic fibrosis and bronchiectasis [4,5]. At these sites, the pathogen forms recalcitrant biofilms and produces virulence factors that contribute to persistent infection, tissue damage, and impaired healing [6,7,8]. Because biofilm-associated tolerance and acquired resistance can limit the effectiveness of conventional antibiotics, complementary antivirulence approaches warrant investigation [9,10,11].

Antivirulence (pathoblocker) approaches aim to reduce pathogenic functions without directly selecting for growth inhibition and may complement antibiotics; however, their effects on resistance evolution must be tested experimentally rather than assumed [12,13]. In *P. aeruginosa*, these programmes are coordinated by quorum sensing (QS), a cell-density-dependent signalling network comprising the interlinked las, rhl and pqs systems that governs biofilm maturation, virulence-factor secretion and motility [14,15]. The las system is mediated by the LuxR-type transcriptional regulator LasR and occupies an upper tier of the canonical QS hierarchy. Accordingly, LasR has been extensively explored as an antivirulence target, and several small-molecule LasR inhibitors have been reported to interfere with QS-regulated phenotypes [16,17,18]. LasR-dependent signalling promotes expression of lasB, which encodes LasB (pseudolysin), a secreted zinc metalloprotease that degrades host elastin, collagen and innate immune effectors and contributes to tissue damage and impaired wound healing [19,20]. Targeting LasB therefore provides a complementary strategy that acts directly on a major downstream virulence effector. Suppression of lasB transcription and direct inhibition of the LasB enzyme are distinct mechanistic objectives and require different experimental evidence.

The recognition of LasB as an antivirulence target has driven sustained medicinal-chemistry efforts to develop small-molecule inhibitors [21]. Early target-based and phenotypic campaigns identified diverse zinc-chelating chemotypes, from phenanthroline-diones to mercapto- and hydroxamate-bearing scaffolds, that suppress LasB activity and protect host cells in Caenorhabditis elegans and cell-based infection models [22,23]. Fragment-based design subsequently afforded thiol inhibitors with well-defined binding modes and early in vivo activity [24], and *N*-aryl mercaptosuccinimide and α-mercaptoacetamide series were optimised to nanomolar potency with improved selectivity over host metalloproteases and efficacy in animal infection models [25,26,27]. Crystallographic studies of these inhibitor complexes have mapped the catalytic zinc, the S1’ specificity pocket and adjacent subsites that govern potency [28], and transition-state-mimicking phosphonates have recently shown in vivo efficacy in a keratitis model [29,30]. Across these chemotypes, however, target engagement rests almost entirely on reversible coordination of the catalytic zinc, which can compromise selectivity and the durability of inhibition and leaves covalent, mechanism-based blockade of LasB largely unexplored.

Medicinal-chemistry optimisation of LasB inhibitors has established an N-aryl/α-isobutyl acetamide as an effective recognition framework, with the para-substituted aryl and α-isobutyl groups occupying the S1’ and S2’ subsites, respectively [27,28,30]. Many reported LasB inhibitors rely on reversible zinc-binding groups, which may present challenges for selectivity against host metalloproteases and pharmacokinetic optimisation [29]. We therefore explored an alternative design in which an epoxide-containing glycidamide motif was introduced to enable potential Glu141-directed covalent engagement, following established principles of electrophile-based covalent inhibitor design [31,32]. Chemical-space mapping of known LasB inhibitors was combined with focused covalent-library enumeration and CovDock-based structural evaluation to guide candidate design and prioritisation [33]. The chemical-space analysis was used to place the glycidamide designs in relation to previously reported LasB inhibitors, whereas final candidate prioritisation was based on the focused structural design and covalent-docking results. **QSI-1** and **QSI-2**, which differ only by a para-CF_3_/para-F substitution, were selected as a matched molecular pair for experimental evaluation. Their antibiofilm and QS-associated activities, direct inhibition of purified LasB, molecular-dynamics behaviour, and combination with azithromycin were subsequently examined, together with the in vivo efficacy and preliminary safety profile of the lead compound. Combination with azithromycin was investigated because antivirulence agents may provide complementary activity when used alongside conventional antibiotics [34].

## 2. Results

### 2.1. Design and Synthesis of **QSI-1** and **QSI-2**

Building on the established N-aryl/α-isobutyl recognition motif of LasB inhibitors [27,28,30], we replaced the conventional reversible zinc-binding group with an epoxide-containing glycidamide motif designed for potential Glu141-directed covalent engagement, following established principles of electrophile-based covalent inhibitor design [31,32]. An initial covalent library was progressively narrowed through focused analogue selection, physicochemical filtering, and Glu141-directed covalent docking, ultimately yielding **QSI-1** and **QSI-2** for synthesis and experimental evaluation (Figure 1A).

To compare the glycidamide design with alternative electrophilic strategies, the epoxide-containing scaffold was evaluated alongside matched non-covalent controls and compounds bearing other electrophilic warheads. Among the tested designs, the glycidamide scaffold gave the most favourable CovDock score for Glu141-directed docking (Figure 1B). The selected glycidamide compounds were subsequently placed in the chemical context of previously reported LasB inhibitors using Morgan fingerprints and t-SNE. In the two-dimensional projection, **QSI-1** and **QSI-2** occupied a region outside that populated by most of the 219 reference LasB bioactivity records (Figure 1C).

The CovDock-derived binding modes of **QSI-1** and **QSI-2** are shown in Figure 1D–G. In these models, Glu141-directed epoxide ring opening generated a modelled covalent ester adduct adjacent to the catalytic Zn^2+^ ion. The glycidamide moiety was positioned toward residues including Asn112, Arg198, and His223, while the leucine isobutyl group occupied the hydrophobic S1’ pocket. The para-substituted aniline extended toward the adjacent lipophilic region, with the 4-CF_3_ substituent of **QSI-1** showing more extensive predicted contacts than the smaller 4-F substituent of **QSI-2**. These binding modes provided a structural basis for comparing the two matched compounds in subsequent experimental assays.

**QSI-1** and **QSI-2** were synthesised through the three-step route shown in Scheme 1 and obtained in yields of 55.0% and 52.5%, respectively. Their structures were confirmed by ^1^H NMR, ^13^C NMR, and HRMS, and both compounds met the purity requirement for subsequent biological evaluation.

### 2.2. Molecular-Dynamics Simulations of the LasB–QSI Complexes

To assess the stability of the CovDock-derived binding modes, 100 ns molecular-dynamics simulations were performed for the LasB–**QSI-1** and LasB–**QSI-2** complexes (Figure 2). The protein backbone remained stable in both systems, with average RMSD values of 0.88 ± 0.08 Å for **QSI-1** and 1.09 ± 0.13 Å for **QSI-2** (Figure 2A). After alignment of the protein backbone, the ligand heavy-atom RMSDs were 1.90 ± 0.26 Å and 2.07 ± 0.56 Å, respectively (Figure 2B). The Cα RMSF profiles were also comparable between the two systems, and the catalytic-site residues His140, Glu141, His144, and Glu164 showed only small fluctuations (<0.46 Å) throughout the simulations (Figure 2C).

A clearer difference was observed in the geometry around the catalytic Zn^2+^ ion. In the **QSI-1** complex, the Zn^2+^–O2 distance remained close to 1.90 ± 0.06 Å throughout the trajectory and stayed below 3.0 Å. In contrast, the corresponding distance in the **QSI-2** complex was larger and more variable (4.24 ± 0.84 Å) and remained above 3.0 Å during the simulation (Figure 2D). The Glu141 Oε2–C7 distance remained stable in both complexes, with average values of 1.460 ± 0.031 Å for **QSI-1** and 1.462 ± 0.032 Å for **QSI-2** (Figure 2E). **QSI-1** also maintained slightly more contacts with surrounding residues than **QSI-2**, averaging 12.21 ± 1.37 and 10.47 ± 1.33 contacting residues per frame, respectively (Figure 2F).

The temperature and system density remained stable throughout the production runs (Figure 2G,H). Taken together, the simulations suggest that both modelled complexes remained structurally stable over 100 ns, while **QSI-1** retained a closer Zn^2+^-proximal geometry and somewhat more extensive local contacts than **QSI-2**.

### 2.3. **QSI-1** and **QSI-2** Inhibit Biofilm Formation at Sub-Micromolar Concentrations

Both **QSI-1** and **QSI-2** reduced *P. aeruginosa* PAO1 biofilm formation in a concentration-dependent manner, decreasing relative biofilm thickness from approximately 100% to 25–30% over the tested concentration range (Figure 3C,D). The calculated IC_50_ values were 0.28 ± 0.03 μM for **QSI-1** and 0.51 ± 0.32 μM for **QSI-2**, corresponding to an approximately 1.8-fold difference in antibiofilm potency. **QSI-1** was therefore selected as the lead compound for subsequent phenotypic evaluation.

### 2.4. **QSI-1** and **QSI-2** Directly Inhibit Purified LasB

To determine whether **QSI-1** and **QSI-2** directly inhibit LasB, their effects on the catalytic activity of purified recombinant LasB were evaluated using the fluorogenic substrate Abz-AGLA-Nba. Both compounds inhibited LasB activity in a concentration-dependent manner (Figure 3E,F), with IC_50_ values of 2.98 ± 0.44 μM for **QSI-1** and 10.20 ± 1.25 μM for **QSI-2**. Thus, **QSI-1** was approximately 3.4-fold more potent than **QSI-2** in the purified-enzyme assay, showing the same directional matched-pair potency trend as observed in the antibiofilm assay.

The structurally related phosphonate LasB inhibitor **4b** was included as a reference compound and displayed nanomolar activity, with an apparent IC_50_ of 0.027 μM (Figure S1), closely consistent with its previously reported IC_50_ of approximately 26 nM. These results support the validity of the biochemical assay and provide direct evidence that **QSI-1** and **QSI-2** inhibit LasB enzymatic activity.

### 2.5. Preferential Reduction in Lasb Promoter Activity by **QSI-1** and **QSI-2**

To examine the effects of **QSI-1** and **QSI-2** on quorum-sensing-associated transcriptional outputs, we used chromosomal GFP reporter strains of *P. aeruginosa* PAO1, including PAO1-*lasB*-*gfp*, PAO1-*rhlA*-*gfp*, and PAO1-*pqsA-gfp*, together with a constitutively expressed PAO1-*gfp* control [33]. Neither **QSI-1** nor **QSI-2** appreciably altered the constitutive GFP signal (Figure 4A,E). Both compounds produced a concentration-dependent reduction in PAO1-*lasB-gfp* reporter activity (Figure 4B,F), whereas the effects on PAO1-*rhlA-gfp* and PAO1-*pqsA-gfp* were more modest (Figure 4C,D,G,H). These results show that **QSI-1** and **QSI-2** preferentially reduce lasB promoter activity relative to the *rhlA* and *pqsA* reporter outputs under the tested conditions.

### 2.6. The Compounds Disarm Biofilms Without Killing the Bacteria

To distinguish biofilm inhibition from bactericidal activity, **QSI-1**-treated biofilms were examined by confocal microscopy after LIVE/DEAD (SYTO 9/PI) staining (Figure 5A). SYTO 9-positive biofilm biomass decreased in a concentration-dependent manner (Figure 5B), whereas PI-positive biomass remained low and did not increase with **QSI-1** treatment (Figure 5C). Consistent with these observations, the number of viable bacteria recovered from the biofilms was not significantly reduced across the tested concentration range (1.25–160 μmol/L; Figure 5D), in contrast to azithromycin, which markedly decreased viable counts. Together with the absence of measurable effects on planktonic growth, these results indicate that **QSI-1** reduces biofilm biomass without exerting detectable bactericidal activity under the tested conditions.

### 2.7. Attenuation of Secreted Virulence Factors

To examine the effects of **QSI-1** on virulence-associated phenotypes, we quantified the production of pyocyanin, rhamnolipid, and exopolysaccharide (EPS). **QSI-1** reduced pyocyanin production in a concentration-dependent manner, with significant inhibition observed from 2.5 μmol/L and greater reductions at higher concentrations (5.0–20 μmol/L; Figure 6A). At the higher concentrations, pyocyanin production was reduced to a level comparable to that observed with azithromycin. In contrast, rhamnolipid and EPS levels remained largely unchanged at lower **QSI-1** concentrations and showed modest reductions only at the higher concentrations tested (20–40 μmol/L; Figure 6B,C). Azithromycin reduced all three measured virulence-associated outputs. Overall, pyocyanin production was more sensitive to **QSI-1** treatment than rhamnolipid or EPS production under the tested conditions.

### 2.8. Inhibition of Surface-Associated Motility

Following the demonstration of reduced virulence-factor production, we investigated the effect of **QSI-1** on bacterial motility phenotypes associated with surface colonisation and biofilm development. **QSI-1** attenuated these behaviours in a phenotype-dependent manner (Figure 7). Swarming motility was the most sensitive, showing a concentration-dependent reduction with significant inhibition from 5 µmol/L and greater effects at 20–40 µmol/L (Figure 7A,D). Swimming motility showed significant reductions from 10 µmol/L (Figure 7B,E), whereas twitching motility was affected only at the highest concentration tested (40 µmol/L; Figure 7C,F). Thus, the three motility phenotypes showed different sensitivities to **QSI-1**, with swarming being the most responsive under the tested conditions.

### 2.9. Potentiation of Azithromycin and In Vivo Efficacy in a Wound-Infection Model

Given the potential of antivirulence agents as adjuncts to conventional antibiotics [34], we examined the effect of combining **QSI-1** with azithromycin against *P. aeruginosa* PAO1 using a checkerboard broth microdilution assay (Figure 8A). **QSI-1** alone did not inhibit planktonic growth within the tested concentration range. In combination, however, **QSI-1** shifted the azithromycin response toward lower concentrations, indicating potentiation of azithromycin activity.

Based on this in vitro potentiation, **QSI-1** was further evaluated in a murine full-thickness excisional wound-infection model (Figure 8B–D). Relative to the vehicle control, topical **QSI-1** treatment (25 μmol/L) accelerated wound closure and produced a modest reduction in local bacterial burden (Figure 8C,D). The combination of **QSI-1** with low-dose azithromycin (0.05 μg/mL) resulted in the greatest improvement in wound closure among the treatment groups and reduced bacterial burden to a level comparable to that observed with the higher azithromycin dose (0.5 μg/mL). These results show that **QSI-1** retains activity in vivo and enhances the therapeutic effect of low-dose azithromycin in this wound-infection model.

### 2.10. **QSI-1** Is Well Tolerated by Mammalian Cells

To define its safety window, **QSI-1** was tested for cytotoxicity against murine RAW264.7 macrophages and human HEK293T cells (Figure 9). **QSI-1** caused no significant loss of RAW264.7 viability at any concentration up to 200 µmol/L (Figure 9A) and only a marginal reduction in HEK293T viability at the two highest concentrations (≥100 μmol/L), with viability remaining above 90% even at 200 µmol/L (Figure 9B). The concentrations associated with detectable cytotoxicity were substantially higher than those required for antibiofilm activity, indicating a favourable preliminary in vitro cytotoxicity profile.

## 3. Discussion

The increasing burden of antimicrobial resistance in *P. aeruginosa* has stimulated interest in antivirulence approaches that attenuate pathogenicity without necessarily inhibiting bacterial growth. Such strategies have been proposed to exert less direct selective pressure than conventional bactericidal or bacteriostatic treatment, although this potential advantage requires experimental evaluation for individual compounds [12,35,36]. Biofilm formation and quorum sensing are central to the persistence and virulence of *P. aeruginosa* [7,8,9], and the QS-controlled metalloprotease LasB is therefore an attractive antivirulence target. Previous medicinal-chemistry studies have established the N-aryl/α-isobutyl motif as an effective recognition framework for LasB inhibitors and have defined the contributions of the S1’ and S2’ binding regions to inhibitor potency [27,28,30]. Building on this structural framework, chemical-space mapping was used to place the glycidamide designs in the context of known LasB inhibitors, while focused covalent-library enumeration and Glu141-directed CovDock analysis were used to prioritise the final candidates. These complementary cheminformatic and structure-based steps provided an integrated framework for the design and selection of **QSI-1** and **QSI-2**.

The biochemical data provide direct evidence that both compounds inhibit LasB enzymatic activity. **QSI-1** inhibited purified recombinant LasB with an IC_50_ of 2.98 ± 0.44 μM, whereas **QSI-2** gave an IC_50_ of 10.20 ± 1.25 μM. The structurally related phosphonate inhibitor **4b** showed nanomolar activity in the same assay, in close agreement with its previously reported LasB IC_50_ of approximately 26 nM [30]. Notably, **QSI-1** was approximately 3.4-fold more potent than **QSI-2** against purified LasB. This trend is consistent with both their antibiofilm activities and the previously reported preference for the 4-CF_3_ analogue over the corresponding 4-F analogue within the phosphonate LasB-inhibitor series [30]. Because this comparison is limited to a single matched pair, we interpret it as a substituent-dependent potency trend rather than a general structure–activity relationship. On the basis of its greater activity in both the antibiofilm and purified-LasB assays, **QSI-1** was advanced as the lead compound for the subsequent phenotypic, combination, and in vivo studies.

The purified-enzyme results also help distinguish direct LasB inhibition from the cellular QS phenotype. Both **QSI-1** and **QSI-2** preferentially reduced lasB promoter activity relative to the rhlA and pqsA reporters, consistent with the organisation of the *P. aeruginosa* QS network [13,14]. At the same time, inhibition of purified LasB demonstrates that their activity is not restricted to transcriptional regulation. The cellular and biochemical results therefore suggest that direct LasB inhibition and an effect at the QS-regulatory level may coexist. The current data do not establish whether the transcriptional effect results from direct LasR modulation or from another upstream regulatory mechanism. This distinction is important because suppression of lasB expression, direct inhibition of LasB enzyme activity, and LasR antagonism represent mechanistically different processes. For **QSI-1**, the reduction in biofilm biomass, selected virulence outputs, and motility without measurable inhibition of planktonic growth is consistent with an antivirulence phenotype [12].

The docking and molecular-dynamics results provide a structural context for the biochemical differences between **QSI-1** and **QSI-2**. The covalent models were constructed with the epoxide positioned toward Glu141, consistent with established principles of electrophile-based covalent inhibitor design [31,32]. During the 100 ns simulations, both modelled complexes remained structurally stable, while **QSI-1** maintained a shorter Zn^2+^–O2 distance and somewhat more extensive local protein contacts than **QSI-2**, consistent with its greater LasB inhibitory activity. Because the Glu141–C7 linkage was predefined in the simulation topology, the MD analysis reflects the persistence of the proposed binding geometry rather than covalent-bond formation.

Previous LasB inhibitors have largely relied on reversible coordination of the catalytic Zn^2+^, including thiol-, hydroxamate-, and phosphonate-containing scaffolds [25,26,27,28,29,30]. The phosphonate inhibitor **4b**, for example, binds the S1’/S2’ region and coordinates the catalytic zinc with nanomolar potency, and has shown activity in several target-validation and infection models [30]. The glycidamide design explored here represents a different approach in which the phosphonate zinc-binding group is replaced by an epoxide-containing motif designed for Glu141-directed engagement, consistent with established principles of electrophile-based covalent inhibitor design [31,32]. Although **4b** remained more potent in the purified-enzyme assay, **QSI-1** and **QSI-2** retained direct LasB inhibitory activity after this scaffold modification. These findings establish the glycidamide series as a chemically distinct LasB-active chemotype and provide a basis for further optimisation of potency and target engagement.

From a translational perspective, **QSI-1** enhanced the activity of azithromycin in the checkerboard assay and improved treatment outcome in the murine wound-infection model when combined with a low azithromycin dose. Antivirulence agents are particularly attractive as adjuncts to conventional antibiotics because attenuation of virulence may facilitate bacterial clearance without requiring strong direct antibacterial activity [4,12]. Cutaneous wounds are also a relevant setting for *P. aeruginosa*, where biofilm formation and virulence-factor production contribute to persistent infection and impaired healing [7,16]. In the present study, **QSI-1** showed no measurable inhibition of planktonic growth and exhibited low cytotoxicity toward RAW264.7 and HEK293T cells at concentrations well above its antibiofilm IC_50_. These findings support the potential of **QSI-1** as an adjunctive antivirulence agent and demonstrate its ability to enhance azithromycin efficacy in the murine wound-infection model.

Several limitations remain. Direct inhibition of purified LasB has now been demonstrated, but the proposed covalent modification of Glu141 has not been experimentally confirmed. Intact-protein mass spectrometry and, ideally, site-resolved LC–MS/MS would provide a more direct test of covalent target engagement. The contribution of LasR or other upstream QS regulators to the reduction in lasB transcription also remains unresolved. In addition, **QSI-1** and **QSI-2** constitute only a single matched pair and are insufficient for a broad SAR analysis. Further work should therefore expand the analogue series, clarify the molecular basis of target engagement, and evaluate activity against a broader panel of clinical *P. aeruginosa* isolates. Pharmacokinetic behaviour, metabolic stability, tissue exposure, and systemic safety will also require further investigation before the therapeutic potential of this chemotype can be assessed. Taken together, the present results identify **QSI-1** and **QSI-2** as covalently designed, LasB-active antivirulence leads and provide a basis for further structural optimisation and mechanistic validation.

## 4. Materials and Methods

### 4.1. Computational Methods

#### 4.1.1. Chemical-Space Visualisation

To place the glycidamide designs in the chemical context of previously reported LasB inhibitors, 219 LasB bioactivity records collected from ChEMBL 36 were represented using 2048-bit radius-2 Morgan fingerprints and projected in two dimensions using t-distributed stochastic neighbour embedding (t-SNE) [33,37]. The projection was used as a qualitative visualisation of the reference chemical space. No supervised machine-learning model was used for activity prediction or for ranking **QSI-1** and **QSI-2**; candidate prioritisation was instead based on the focused covalent-library design and CovDock analysis described below.

#### 4.1.2. Covalent Docking and Binding-Mode Analysis

The LasB structure (PDB ID: 8CC4) was prepared using the Protein Preparation Wizard implemented in Maestro 12.8 (Schrödinger, LLC, New York, NY, USA). Bond orders and formal charges were assigned, hydrogen atoms were added, and protonation states were determined at pH 7.4 using PROPKA. The catalytic Zn^2+^ ion and its His140/His144/Glu164 coordination environment were retained, and the protein structure was restrained-minimised using the OPLS4 force field. Glu141 was modelled in its deprotonated carboxylate form, and ligands were prepared using LigPrep with OPLS4.

Covalent docking was performed using CovDock with Glu141 specified as the reactive residue. For the glycidamide compounds, epoxide ring opening was selected as the covalent reaction [32], generating a Glu141-linked adduct for pose evaluation. Poses were ranked according to the CovDock score. To compare the glycidamide design with alternative reactive motifs, matched non-covalent controls and a panel of alternative electrophilic warheads were evaluated using the corresponding docking protocols. The resulting poses were inspected for interactions with the catalytic Zn^2+^ region and the S1’/S2’ binding subsites.

#### 4.1.3. Molecular Dynamics Simulations

The CovDock-derived LasB–**QSI-1** and LasB–**QSI-2** complexes were used as starting structures for molecular dynamics (MD) simulations. Simulations were performed using OpenMM 8.3.1 with the Amber ff14SB force field for LasB and GAFF2 parameters for the ligands. The Glu141 Oε2–ligand C7 linkage was explicitly defined in the topology using parameters derived from chemically analogous GAFF2 terms. Each complex was solvated in a periodic TIP3P water box with a 10 Å buffer and approximately 0.15 M NaCl. Long-range electrostatic interactions were treated using the particle-mesh Ewald method.

The native coordination geometries of the catalytic Zn^2+^ ion and structural Ca^2+^ ion were maintained using harmonic distance restraints, whereas no restraint was applied between Zn^2+^ and ligand O2. Following energy minimisation and 0.5 ns of staged equilibration, a single 100 ns production trajectory was generated for each complex in the NPT ensemble at 300 K and 1.01325 bar using a 2 fs time step. Coordinates were saved every 100 ps.

Trajectory analysis was performed using MDTraj 1.11.1. Protein-backbone RMSD, ligand heavy-atom RMSD, Cα RMSF, Zn^2+^–ligand O2 distance, Glu141 Oε2–ligand C7 distance, and protein–ligand contacts within 4.0 Å were calculated from the production trajectories. The covalently linked Glu141 residue was excluded from the non-covalent contact analysis. Temperature and system density were also monitored throughout the simulations. Numerical summaries were calculated from the raw trajectories, whereas approximately 1 ns rolling averages were used only for graphical presentation.

### 4.2. Chemistry

#### 4.2.1. General Chemistry

All reagents and anhydrous solvents were commercially purchased from standard suppliers, including Bide Pharmatech Co., Ltd (Shanghai, China)and Energy Chemical (Shanghai, China), and were used without further purification unless otherwise stated. Syntheses were performed in accordance with Scheme 1, and the reactions were monitored by thin-layer chromatography (TLC) and liquid chromatography-mass spectrometry (LC-MS). Final products were purified via flash column chromatography. ^1^H NMR and ^13^C NMR spectra were recorded using a Bruker DRX-400 spectrometer (400 MHz; Bruker BioSpin GmbH, Rheinstetten, Germany). The products were dissolved in deuterated chloroform (CDCl_3_), and chemical shifts were referenced to the residual solvent peaks. High-resolution mass spectrometry (HRMS) data were acquired utilising either a Finnigan MAT-90 mass spectrometer (Finnigan MAT GmbH, Bremen, Germany) or an Agilent 6210 LC/MSD TOF mass spectrometer (Agilent Technologies, Santa Clara, CA, USA). Samples were dissolved in chromatography-grade methanol, filtered through a 0.22 μm membrane, and analysed in electrospray ionisation (ESI) positive mode Analytical purity was determined using a Thermo Scientific Dionex Ultimate 3000 high-performance liquid chromatography (HPLC) system (Thermo Fisher Scientific, Waltham, MA, USA) equipped with a reversed-phase C18 column, using chromatography-grade methanol and ultrapure water as the mobile phase. All final compounds subjected to biological evaluation demonstrated an HPLC purity of ≥95%. The reference LasB inhibitor **4b** was synthesised according to the previously reported procedure [30], and its identity and purity were confirmed before use in the enzymatic assay.

#### 4.2.2. Synthesis of **QSI-1** and **QSI-2**

The target compounds were synthesised as outlined in Scheme 1. First, *N*-Boc-L-leucine was coupled with either 4-(trifluoromethyl)aniline (for **QSI-1**) or 4-fluoroaniline (for **QSI-2**) using HATU and DIPEA in DMF, generating the corresponding *N*-Boc leucine anilides. Subsequent removal of the Boc protecting group was achieved using trifluoroacetic acid in dichloromethane. The resulting free α-amino anilide intermediates were then acylated with potassium oxirane-2-carboxylate (potassium glycidate) in the presence of HATU in DMF. This final step installed the reactive epoxide (glycidamide) warhead, affording the desired final compounds **QSI-1** (R = CF_3_) and **QSI-2** (R = F).

#### 4.2.3. *N*-(4-Methyl-1-oxo-1-((4-(trifluoromethyl)phenyl)amino)pentan-2-yl)oxirane-2-carboxamide (**QSI-1**)

To a stirred solution of leucine 4-(trifluoromethyl)anilide (274.3 mg, 1.0 mmol) and potassium oxirane-2-carboxylate (potassium glycidate, 138.8 mg, 1.1 mmol) in dry DMF (5.0 mL) under an argon atmosphere, HATU (456.3 mg, 1.2 mmol) and DIPEA (0.52 mL, 3.0 mmol) were added at 0 °C. The reaction mixture was allowed to warm to room temperature and stirred for 6 h. Upon completion, the mixture was diluted with water and extracted with ethyl acetate. The combined organic layers were washed with brine, dried over anhydrous Na_2_SO_4_, and concentrated under reduced pressure. Purification of the crude residue by column chromatography (petroleum ether/ethyl acetate = 5:1) afforded **QSI-1** as white solid material (189.4 mg, 55.0% yield); its crystalline or amorphous form was not determined. ^1^H NMR (400 MHz, CDCl3) *δ* 9.03/9.01 (s, 1H), 7.68 − 7.52 (m, 4H), 6.74 − 6.66 (m, 1H), 4.70 − 4.61 (m, 1H), 3.54 − 3.51 (m, 1H), 3.09 − 2.81 (m, 2H), 1.85 − 1.59 (m, 3H), 0.99 − 0.90 (m, 6H). ^13^C NMR (101 MHz, CDCl_3_) *δ* 169.7, 140.8, 126.2 (q, *J* = 32.0 Hz), 124.3 (q, *J* = 271.5 Hz), 119.5, 119.4, 52.1, 49.3, 47.7, 40.4, 24.8, 22.8, 21.9. HRMS (ESI) of compound **QSI-1**: calcd for C_16_H_20_F_3_N_2_O_3_ [M + H]^+^ = 345.1421, found [M + H]^+^ = 345.1415.

#### 4.2.4. *N*-(1-((4-Fluorophenyl)amino)-4-methyl-1-oxopentan-2-yl)oxirane-2-carboxamide (**QSI-2**)

Following the general coupling procedure described for **QSI-1**, the reaction of leucine 4-fluoroanilide with potassium glycidate yielded **QSI-2** as white solid material (154.5 mg, 52.5% yield); its crystalline or amorphous form was not determined. ^1^H NMR (400 MHz, CDCl3) *δ* 8.99 (s, 1H), 7.53 − 7.45 (m, 2H), 7.02 − 6.94 (m, 2H), 4.74 − 4.66 (m, 1H), 3.51 − 3.47 (m, 1H), 3.03 − 2.78 (m, 2H), 1.80 − 1.58 (m, 3H), 0.97 − 0.88 (m, 6H). ^13^C NMR (101 MHz, CDCl_3_) *δ* 169.9, 169.4, 159.4 (d, *J* = 243.5 Hz), 133.8, 121.7 (d, *J* = 7.8 Hz), 115.6 (d, *J* = 22.5 Hz), 52.0, 49.4, 47.5, 41.1, 24.9, 22.8, 22.0. HRMS (ESI) of compound **QSI-2**: calcd for C_15_H_20_FN_2_O_3_ [M + H]^+^ = 295.1453, found [M + H]^+^ = 295.1448.

### 4.3. Bacterial Growth-Curve Analysis

To confirm a disarming rather than bactericidal mode of action, *P. aeruginosa* PAO1 was inoculated into ABTGC medium (initial OD_600_ = 0.02) with **QSI-1** across the tested concentration range. Suspensions (200 µL/well) in 96-well plates were monitored at 37 °C for 24 h, with OD_600_ recorded every 10 min using a Varioskan LUX multimode microplate reader (Thermo Fisher Scientific, Singapore). Experiments were performed in triplicate.

### 4.4. Biofilm Assays

#### 4.4.1. Biofilm Formation Assay

Biofilm formation was quantified by crystal-violet staining. *P. aeruginosa* PAO1 was standardised to OD_600_ = 0.02 and co-incubated with different concentrations of **QSI-1** in biofilm-inducing ABTGC medium (B-medium [0.1% MgCl_2_, 0.1% CaCl_2_, 0.1% FeCl_3_] supplemented with 10% (*v*/*v*) A10, 0.2% D-glucose and 0.2% casamino acids) in 96-well plates (200 µL/well). 0.1% (*v*/*v*) DMSO and azithromycin served as negative and positive controls, respectively. After 24 h of static incubation at 37 °C, biofilms were washed three times with PBS (3 × 300 µL), fixed with methanol (150 µL, 30 min) and stained with 0.1% crystal violet (30 min). Excess dye was removed by rinsing with distilled water, and the bound dye was solubilized in 33% (*v*/*v*) acetic acid (150 µL). Absorbance was measured at 570 nm (BioTek microplate reader).

#### 4.4.2. Biofilm Architecture and Viability (Confocal Laser-Scanning Microscopy)

*P. aeruginosa* PAO1 biofilms were cultured in glass-bottom dishes in ABTGC medium containing **QSI-1** (0.1–40 µmol/L) for 24 h, gently washed with PBS, and dual-stained with SYTO 9 (1 µmol/L, viable cells) and propidium iodide (10 µmol/L, dead cells) for 15 min. Fluorescence z-stacks were acquired using an LSM800confocal laser-scanning microscope (Carl Zeiss Microscopy GmbH, Jena, Germany) microscope with a 63× oil-immersion objective. Biofilm thickness and live and dead biomass were quantified usingZen 3.10 software (Carl Zeiss Microscopy GmbH, Jena, Germany) relative to DMSO controls in biological triplicate. Relative biofilm-thickness concentration-response data (Figure 3C,D) were fitted by nonlinear regression to obtain IC_50_ values.

#### 4.4.3. Biofilm Viability

Viable bacteria within biofilms were enumerated by colony counting. Biofilms were cultivated in 6-well plates as described above and treated with **QSI-1** (1.25–160 μmol/L) for 24 h. The medium was removed, and the biofilms were gently washed with sterile PBS to remove planktonic cells. Biofilm-associated bacteria were detached by scraping and vigorous pipetting in 1 mL PBS, followed by sonication (5 min, 40 kHz) to disrupt aggregates. The suspensions were serially diluted, plated on LB agar, and incubated at 37 °C for 18 h. Colonies were counted and expressed as log_10_ CFU/cm^2^.

### 4.5. Purified LasB Enzymatic Inhibition Assay

The direct inhibitory effects of **QSI-1** and **QSI-2** on LasB enzymatic activity were evaluated using a fluorogenic substrate-based assay adapted from Everett et al. [26]. Recombinant *P*. *aeruginosa* LasB (10 ng/well) was incubated in 50 mM Tris-HCl (pH 7.4) containing 2.5 mM CaCl_2_ and 0.01% (*v*/*v*) Triton X-100. **QSI-1** and **QSI-2** (0.003–100 μM) or the reference LasB inhibitor **4b** were pre-incubated with LasB for 30 min, followed by addition of the fluorogenic substrate Abz-AGLA-Nba (20 μM) to initiate the reaction. The final reaction volume was 100 μL, with a constant DMSO concentration across all wells.

Fluorescence was monitored kinetically using a Varioskan LUX multimode microplate reader (Life Technologies Holdings Pte. Ltd., Thermo Fisher Scientific, Singapore) at excitation/emission wavelengths of 315/430 nm. Initial reaction rates were calculated from the linear portion of the fluorescence-time curves and normalised to the vehicle control to determine LasB inhibition. Appropriate substrate and compound blanks were included for background correction and assessment of fluorescence interference. Concentration-response curves were fitted using a four-parameter variable-slope model in GraphPad Prism 9.3.1 (GraphPad Software, Inc., San Diego, CA, USA) to determine IC_50_ values. Three independent experiments were performed, with each condition measured in technical triplicate, and IC_50_ values are presented as mean ± SD.

### 4.6. Bacterial Strains and Growth Conditions

*P. aeruginosa* PAO1 (ATCC 15692) and its isogenic GFP transcriptional-reporter derivatives PAO1-*gfp* (constitutive control), PAO1-*lasB-gfp*, PAO1-*rhlA-gfp*, and PAO1-*pqsA-gfp* were used. Stocks were cryopreserved in LB broth containing 25% (*v/v*) glycerol at −80 °C. For each experiment, strains were revived on LB agar (1.5% *w/v*) for 18 h at 37 °C, and single colonies were cultured in LB broth to stationary phase (16 h, 37 °C, 200 rpm). Cell densities were standardised spectrophotometrically by OD_600_.

For GFP transcriptional-reporter assays, overnight cultures were diluted in ABTGC medium to an initial OD_600_ of 0.05 and treated with QSI-1 or QSI-2 at 1.25, 2.5, 5.0, and 10 μmol/L, with vehicle-treated cultures serving as controls. Bacterial suspensions were transferred to sterile black-walled 96-well plates and incubated at 37 °C. GFP fluorescence (excitation, 485 nm; emission, 528 nm) and OD_600_ were recorded every 15 min for at least 16 h using a BioTek multimode microplate reader. Three independent biological experiments were performed for each condition.

### 4.7. Virulence-Factor Quantification

Cultures were prepared in LB medium (initial OD600 = 0.01) and treated with QSI-1 (0–40 μmol/L) or azithromycin (10 μmol/L; positive control) for 24 h at 37 °C and 200 rpm, using 0.1% (*v/v*) DMSO as the vehicle (negative) control. These assays comprised three independent biological culture replicates; four technical wells were averaged within each biological replicate and were not counted as independent n. The effects of QSI-1 and azithromycin on virulence-factor production were expressed as percentages relative to the vehicle control, calculated using the following equation:

$$Relative\;Production\;(\%)=\frac{{OD}_{trested}}{{OD}_{control}}\times 100$$

#### 4.7.1. Pyocyanin

Cell-free supernatant (6 mL; 10,000× g, 5 min, 4 °C) was extracted with chloroform (1:0.25 *v/v*); the organic phase (1.0 mL) was acidified with 0.2 mol/L HCl (0.3 mL), and after phase-inversion centrifugation (10,000× *g*, 5 min) the chromogenic aqueous layer was measured at 520 nm.

#### 4.7.2. Rhamnolipid

Supernatant (2 mL) was extracted with diethyl ether (1:2 *v*/*v*); the evaporated residue was reacted with 0.19% (*w*/*v*) orcinol in 50% H_2_SO_4_ at 80 °C for 30 min, and the product measured at 421 nm, normalised to growth density.

#### 4.7.3. Exopolysaccharide (EPS)

Cell-free supernatant (5 mL) was mixed with chilled absolute ethanol (1:3 *v*/*v*) and incubated at 4 °C for 24 h; precipitated EPS was collected (12,000× g, 10 min, 4 °C), washed twice with cold ethanol, vacuum-dried (50 °C, 2 h) and reconstituted in ultrapure water (5 mL). Aliquots (1 mL) were reacted with 5% (*w/v*) phenol (0.5 mL) and concentrated H_2_SO_4_ (2.5 mL), incubated at 90 °C for 30 min, and the absorbance was measured at 490 nm.

### 4.8. Motility Assays

To evaluate the effects of **QSI-1** on bacterial motility, swarming, swimming, and twitching assays were performed using specific agar plates containing the compound (0–40 µmol/L) or vehicle control (0.1% *v*/*v* DMSO). For the swimming motility assay, plates containing LB broth supplemented with 0.3% (*w*/*v*) agar were spot-inoculated at the centre with 2 µL of bacterial suspension (OD_600_ = 1.0) and incubated upright at 37 °C for 24 h. For the swarming motility assay, plates composed of nutrient broth supplemented with 0.5% (*w*/*v*) agar and 0.5% (*w*/*v*) glucose were surface-inoculated with 2 µL of the bacterial suspension and incubated at 37 °C for 24 h. For the twitching motility assay, LB plates containing 1.0% (*w*/*v*) agar were stab-inoculated with a sterile toothpick through the agar layer to the bottom of the Petri dish; after incubation at 37 °C for 24–48 h, the agar layer was carefully discarded, and the bacterial adherence zone on the plastic surface was stained with 1% (*w*/*v*) crystal violet for visualisation. All assays were performed in triplicate, and motility was quantified by measuring the diameters (mm) of the respective migration zones.

### 4.9. Checkerboard Combination Assay

The effect of combining **QSI-1** with azithromycin was examined by checkerboard broth microdilution. PAO1 (OD_600_ = 0.05) was exposed to serial dilutions of **QSI-1** and antibiotic in 96-well plates (100 µL each; 24 h, 37 °C, static), and growth was read at OD600. A classical fractional inhibitory concentration index requires finite single-agent MIC values for both components. Because **QSI-1** did not reach a single-agent MIC within the tested range, its fractional inhibitory concentration and hence the FICI were undefined. The combination was therefore summarised descriptively by the change in the apparent azithromycin MIC_50_ at a fixed **QSI-1** concentration, without assigning a formal synergy category.

### 4.10. In Vivo Wound-Infection Study

All animal procedures were approved by the Experimental Animal Ethics Committee of Jinan University and conducted in compliance with China’s National Regulations on Animal Experimentation (GB/T 35892-2018). A murine full-thickness excisional wound-infection model was established using female BALB/c mice (3–5 weeks old; specific-pathogen-free). Six mice were included in each experimental group, consistent with our previously published study using a comparable *P. aeruginosa* PAO1 murine wound-infection model [33]. The individual mouse was considered the experimental unit.

Mice were anaesthetised with 4% chloral hydrate (400 mg/kg, i.p.), and a 5.0 ± 0.2 mm full-thickness wound was created on the depilated dorsum using a sterile 5 mm biopsy punch. Each wound was topically inoculated with 30 µL of *P. aeruginosa* PAO1 (5 × 10^8^ CFU/mL in PBS). Beginning 24 h post-infection, topical treatment (30 µL per wound) was administered once daily to five groups: vehicle control (saline); high-dose azithromycin (0.5 µg/mL); low-dose azithromycin (0.05 µg/mL); QSI-1 alone (25 µmol/L); and QSI-1 (25 µmol/L) combined with low-dose azithromycin (0.05 µg/mL). Based on the 30 µL application volume and an average body weight of 20 g, the corresponding administered doses were approximately 0.013 mg/kg for QSI-1 (25 µmol/L), 0.00075 mg/kg for azithromycin (0.5 µg/mL), and 0.000075 mg/kg for azithromycin (0.05 µg/mL).

General condition and body weight were monitored daily, and wounds were photographed at the indicated time points to monitor healing. Outcomes were assessed over 7 days under blinded conditions and included wound closure, with wound area expressed as a percentage of the day 0 value, and local bacterial burden, expressed as log10 CFU per wound. Bacterial burden was determined at the indicated time points by quantitative culture of wound-swab samples following serial dilution and plating.

### 4.11. Statistical Analysis

Unless otherwise stated, cell-based in vitro experiments were performed in three independent biological replicates (n = 3), with technical replicates averaged within each experiment, and in vivo experiments were performed with six animals per group (n = 6). Data are presented as mean ± SD. Statistical analyses were performed using GraphPad Prism 9.3.1. Comparisons were conducted using one- or two-way ANOVA followed by Šídák’s multiple-comparisons test, as appropriate. *p* < 0.05 was considered statistically significant.

## 5. Conclusions

This study reports the structure-guided design and biological evaluation of the glycidamido-leucinamide derivatives **QSI-1** and **QSI-2** as LasB-active antivirulence agents against *Pseudomonas aeruginosa*. Both compounds directly inhibited purified LasB and suppressed biofilm formation, with **QSI-1** showing greater activity than **QSI-2** in both assays.**QSI-1** further reduced *lasB* promoter activity, attenuated virulence-factor production and surface-associated motility, and showed no measurable inhibition of planktonic bacterial growth under the tested conditions. In addition, **QSI-1** potentiated the activity of azithromycin in vitro, while its combination with low-dose azithromycin was associated with improved wound closure and reduced local bacterial burden in the murine wound-infection model. Together, these findings establish the glycidamido-leucinamide scaffold as a promising basis for the further development of LasB-targeted antivirulence agents and support **QSI-1** as a lead compound for subsequent optimisation and mechanistic investigation.

## Figures and Tables

**Figure 1:**
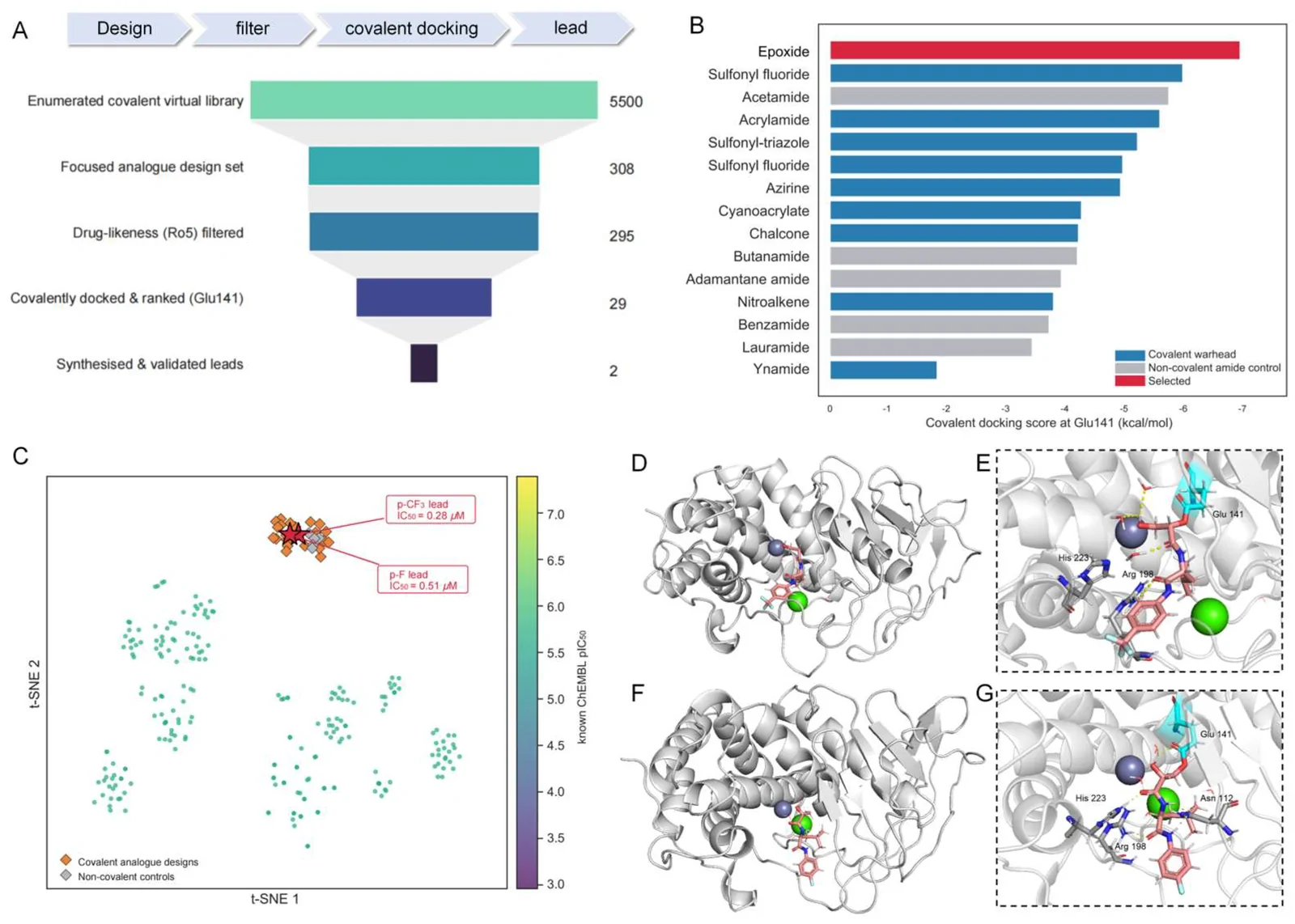
Computational design workflow and predicted binding modes of **QSI-1** and **QSI-2**. (**A**) Screening workflow used to prioritise compounds from the focused covalent library. (**B**) Comparison of CovDock scores for Glu141-directed designs bearing different electrophilic warheads and matched non-covalent controls. (**C**) Morgan-fingerprint-based t-SNE projection of the designed glycidamides and 219 reported LasB bioactivity records, with reference compounds coloured by pIC_50_. (**D**,**E**) Predicted covalent docking pose of **QSI-1** and (**F**,**G**) that of **QSI-2** in the LasB active site.

**Figure 2:**
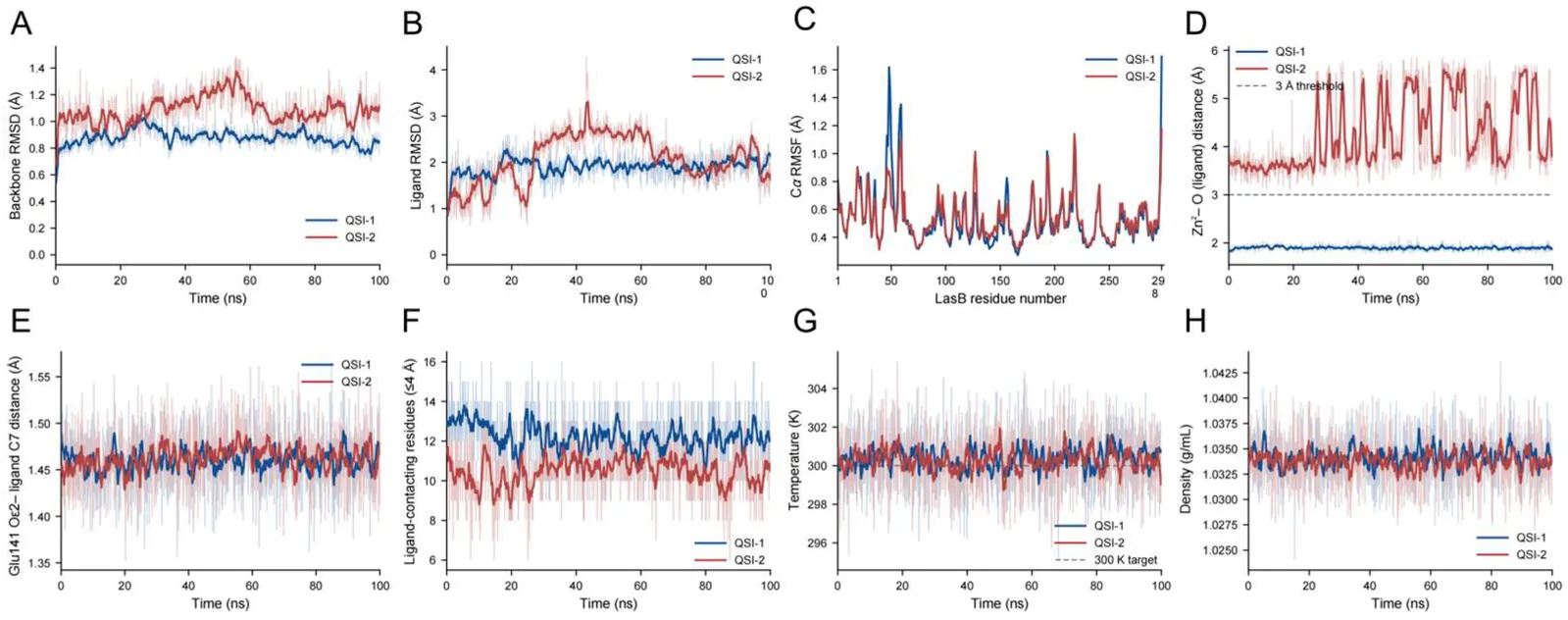
Structural persistence and interaction geometry during 100 ns molecular-dynamics simulations of the explicitly bonded LasB–**QSI-1** and LasB–**QSI-2** models. (**A**) Protein-backbone RMSD after fitting to the first production frame. (**B**) Ligand heavy-atom RMSD calculated after the same protein fit. (**C**) C*α* RMSF over LasB residues 1–298. (**D**) Unrestrained distance between the catalytic Zn ion and ligand O2 atom; the dashed line denotes the operational 3 Å threshold. (**E**) Distance between the explicitly bonded Glu141 Oε2 and ligand C7 atoms. (**F**) Number of non-covalently contacting protein residues within 4 Å of the ligand; the covalently linked Glu141 residue was excluded. (**G**) Production temperature; the dashed line denotes the 300 K target. (**H**) System density. Pale lines show the raw saved values, whereas dark lines show centred approximately 1 ns rolling means, except in panel (**C**), which presents residue-level RMSF profiles. Values were obtained from one trajectory per model and therefore represent temporal variation rather than independent-replicate uncertainty.

**Figure 3:**
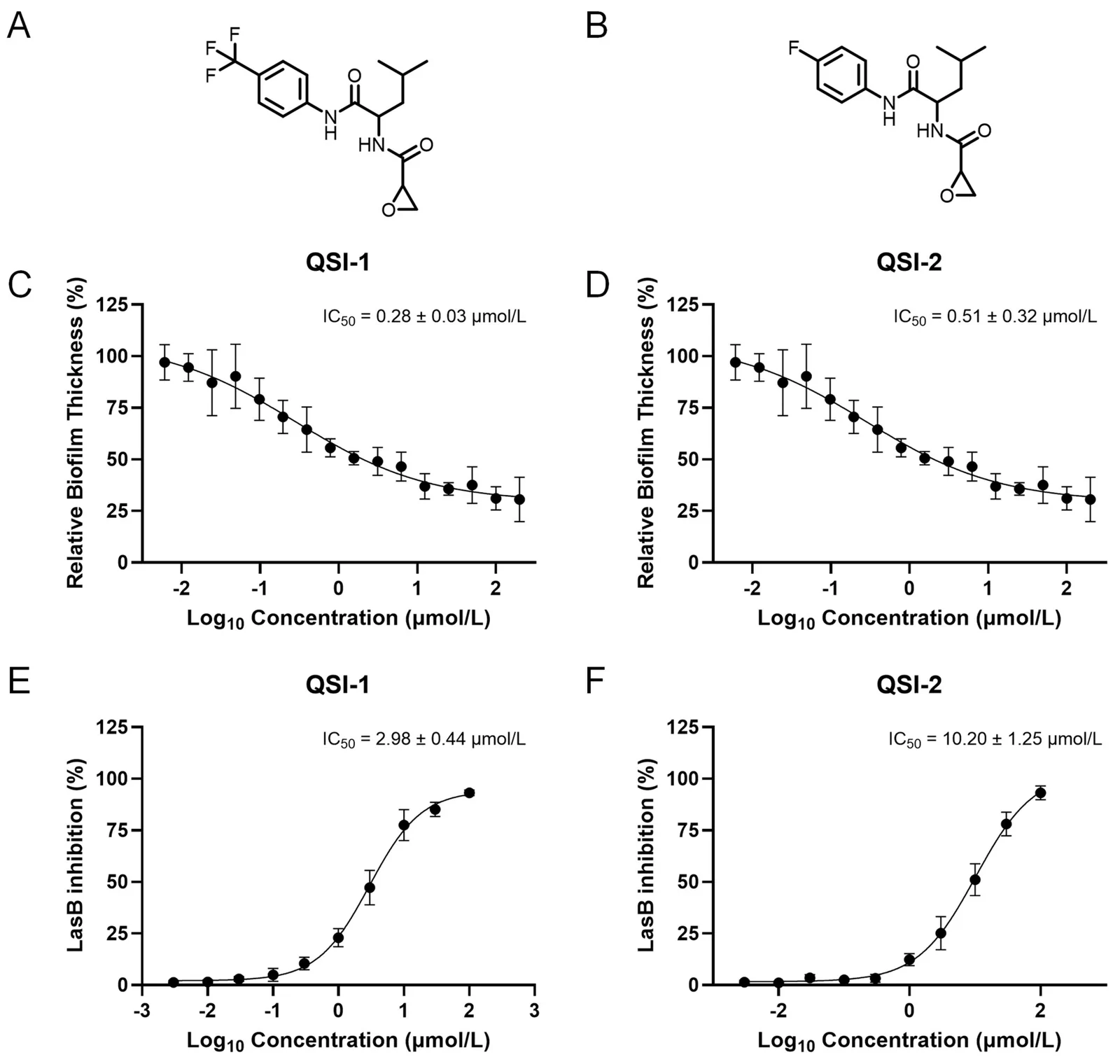
Structures, antibiofilm activity, and direct LasB inhibition of **QSI-1** and **QSI-2**. (**A**,**B**) Chemical structures of **QSI-1** (4-CF_3_) and **QSI-2** (4-F). (**C**,**D**) Concentration-dependent effects of **QSI-1** and **QSI-2** on the relative biofilm thickness of *P. aeruginosa* PAO1, with IC_50_ values of 0.28 ± 0.03 and 0.51 ± 0.32 μM, respectively. (**E**,**F**) Concentration-dependent inhibition of purified recombinant LasB by **QSI-1** and **QSI-2**, with IC_50_ values of 2.98 ± 0.44 and 10.20 ± 1.25 μM, respectively. LasB activity was measured using the fluorogenic substrate Abz-AGLA-Nba. Data are presented as mean ± SD from three independent experiments, and concentration–response curves were fitted using a four-parameter variable-slope model.

**Figure 4:**
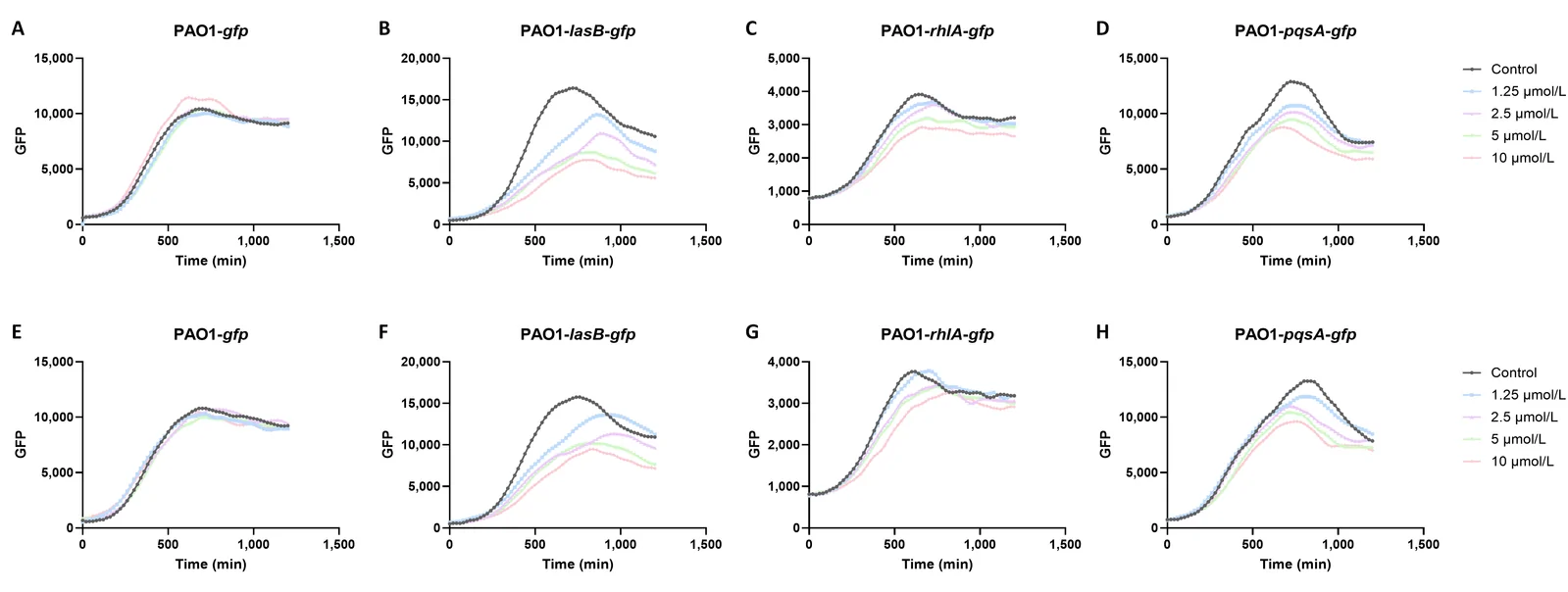
Quorum-sensing reporter activity. GFP output over time for PAO1-*gfp* (constitutive control), PAO1-*lasB-gfp*, PAO1-*rhlA-gfp*, and PAO1-*pqsA-gfp* treated with **QSI-1** (**A**–**D**) or **QSI-2** (**E**–**H**) at 1.25–10 μmol/L versus vehicle control.

**Figure 5:**
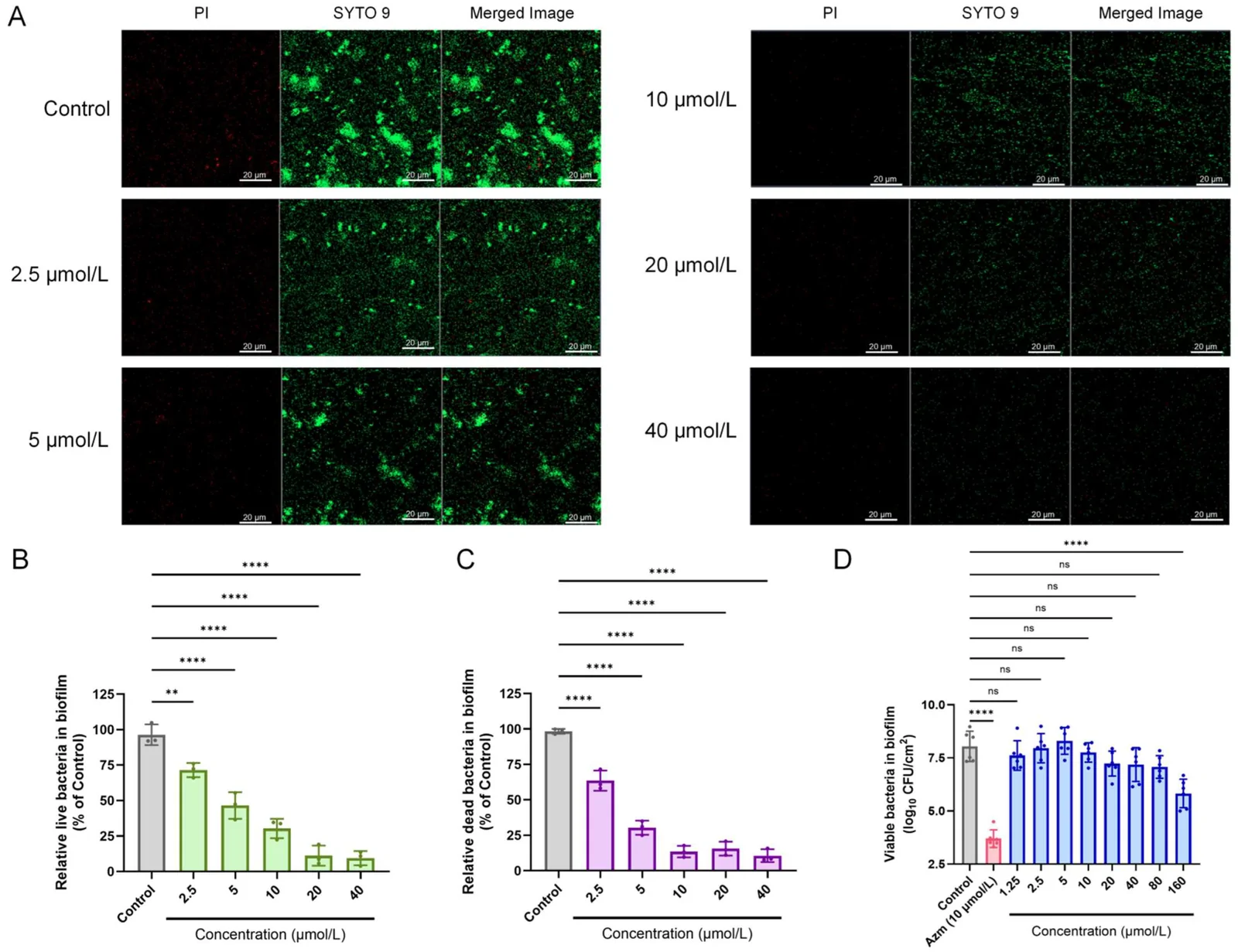
Biofilm architecture, viability, and non-bactericidal activity of **QSI-1** (**A**) Representative CLSM images of PAO1 biofilms following treatment with **QSI-1** (0–40 μmol/L). PI fluorescence is shown in red and indicates dead cells, whereas SYTO 9 fluorescence is shown in green and indicates live cells; merged images show the superimposition of the two fluorescence channels. (**B**,**C**) Relative SYTO 9-positive live biofilm biomass and PI-positive dead biofilm biomass, respectively, expressed as percentages of the vehicle control. Green and purple bars represent live and dead biofilm biomass, respectively. (**D**) Viable bacteria recovered from biofilms (log_10_ CFU/cm^2^) following **QSI-1** treatment (1.25–160 μmol/L); gray indicates the vehicle control, red indicates azithromycin (Azm, 10 μmol/L; positive control), and blue indicates QSI-1-treated groups.. Data are presented as mean ± SD. ** *p* < 0.01, **** *p* < 0.0001; ns, not significant.

**Figure 6:**
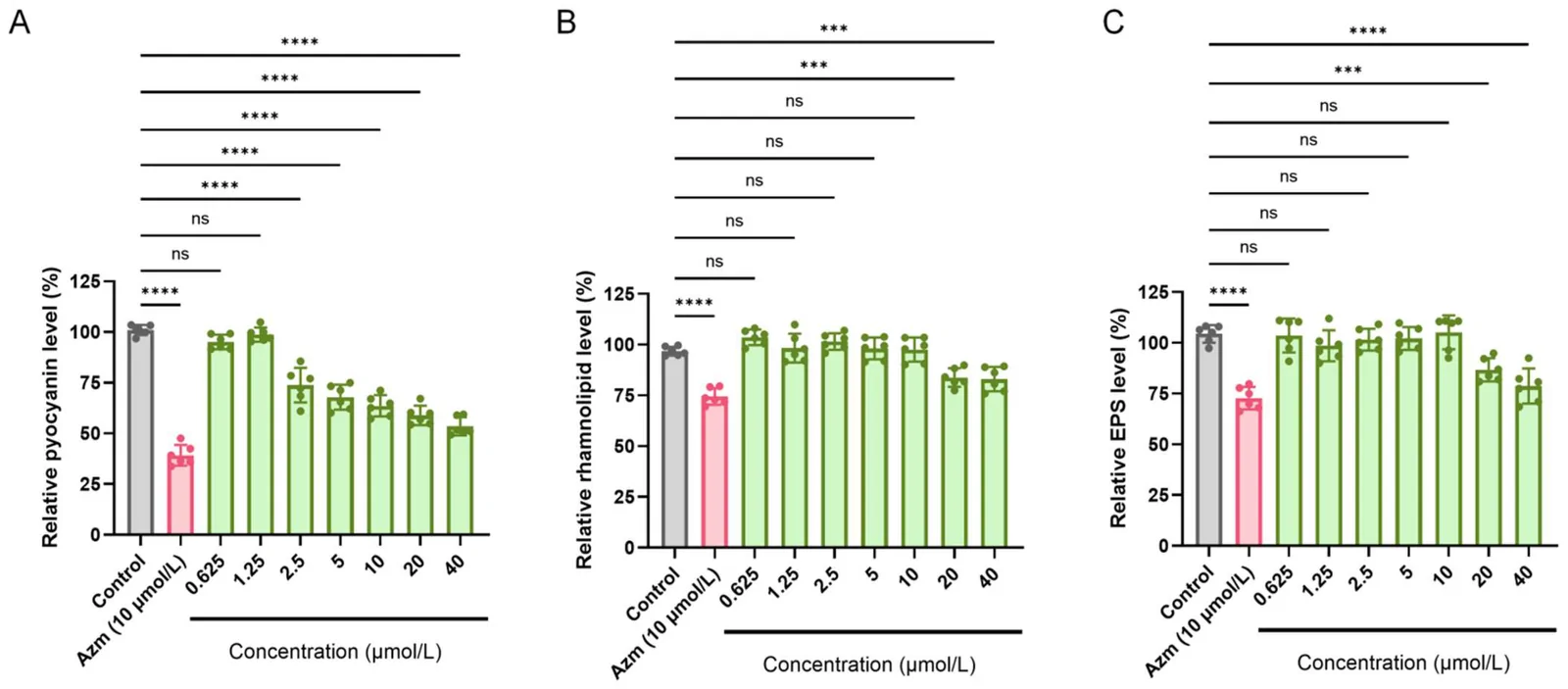
Effects of **QSI-1** on virulence-factor production. Relative levels of (**A**) pyocyanin, (**B**) rhamnolipid, and (**C**) EPS following **QSI-1** treatment; azithromycin (Azm, 10 μmol/L) was included as a positive control.Gray bars indicate the vehicle control, the red bar indicates azithromycin, and green bars indicate **QSI-1**-treated groups. Data are expressed relative to the vehicle control. *** *p* < 0.001, **** *p* < 0.0001; ns, not significant.

**Figure 7:**
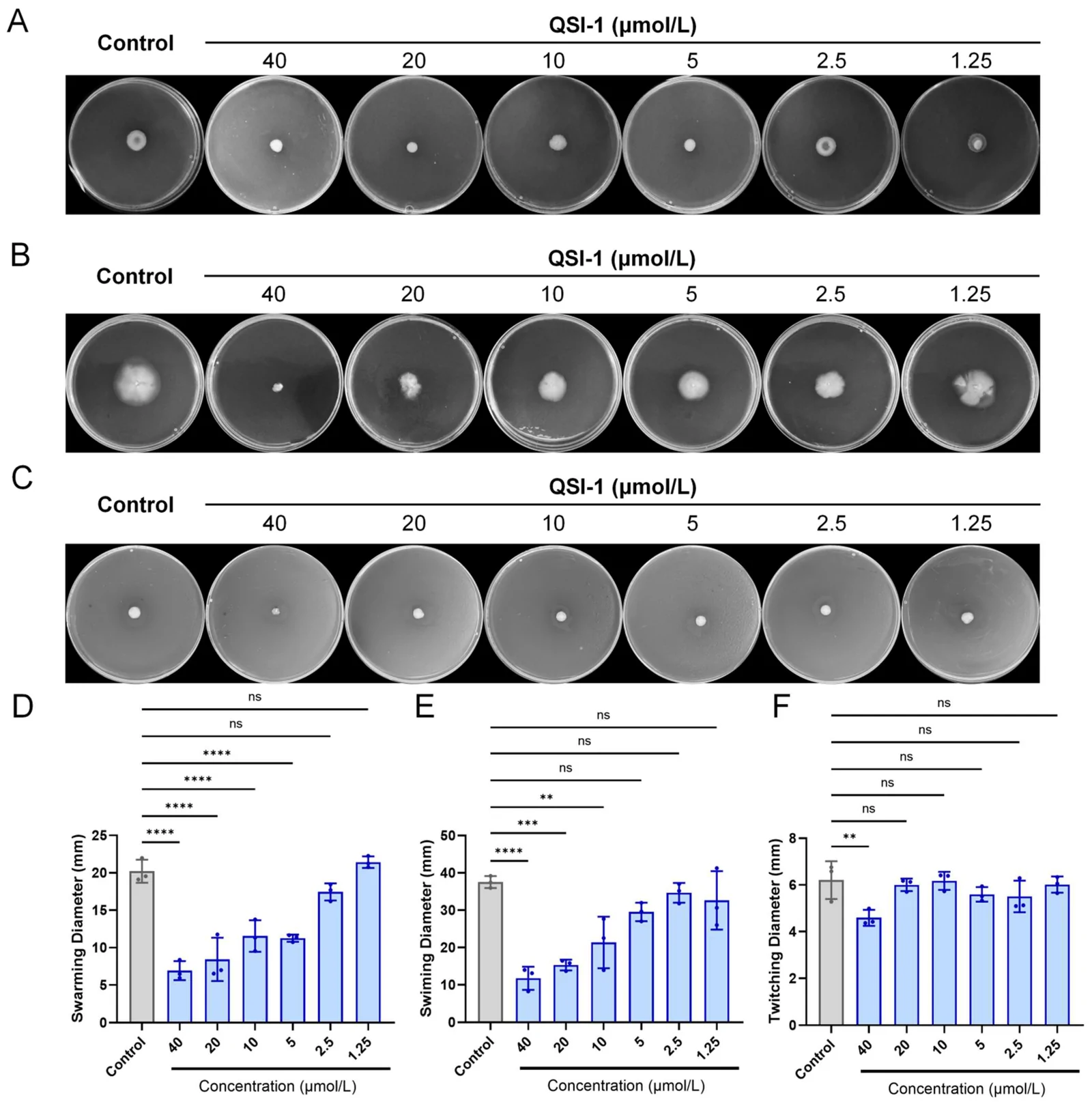
Effects of **QSI-1** on surface-associated motility. Representative plates and quantification of (**A**,**D**) swarming, (**B**,**E**) swimming, and (**C**,**F**) twitching motility following treatment with QSI-1 at 1.25–40 µmol/L. Gray bars indicate the vehicle control, and blue bars indicate QSI-1-treated groups. Data are presented as mean ± SD. ** *p* < 0.01, *** *p* < 0.001, **** *p* < 0.0001; ns, not significant.

**Figure 8:**
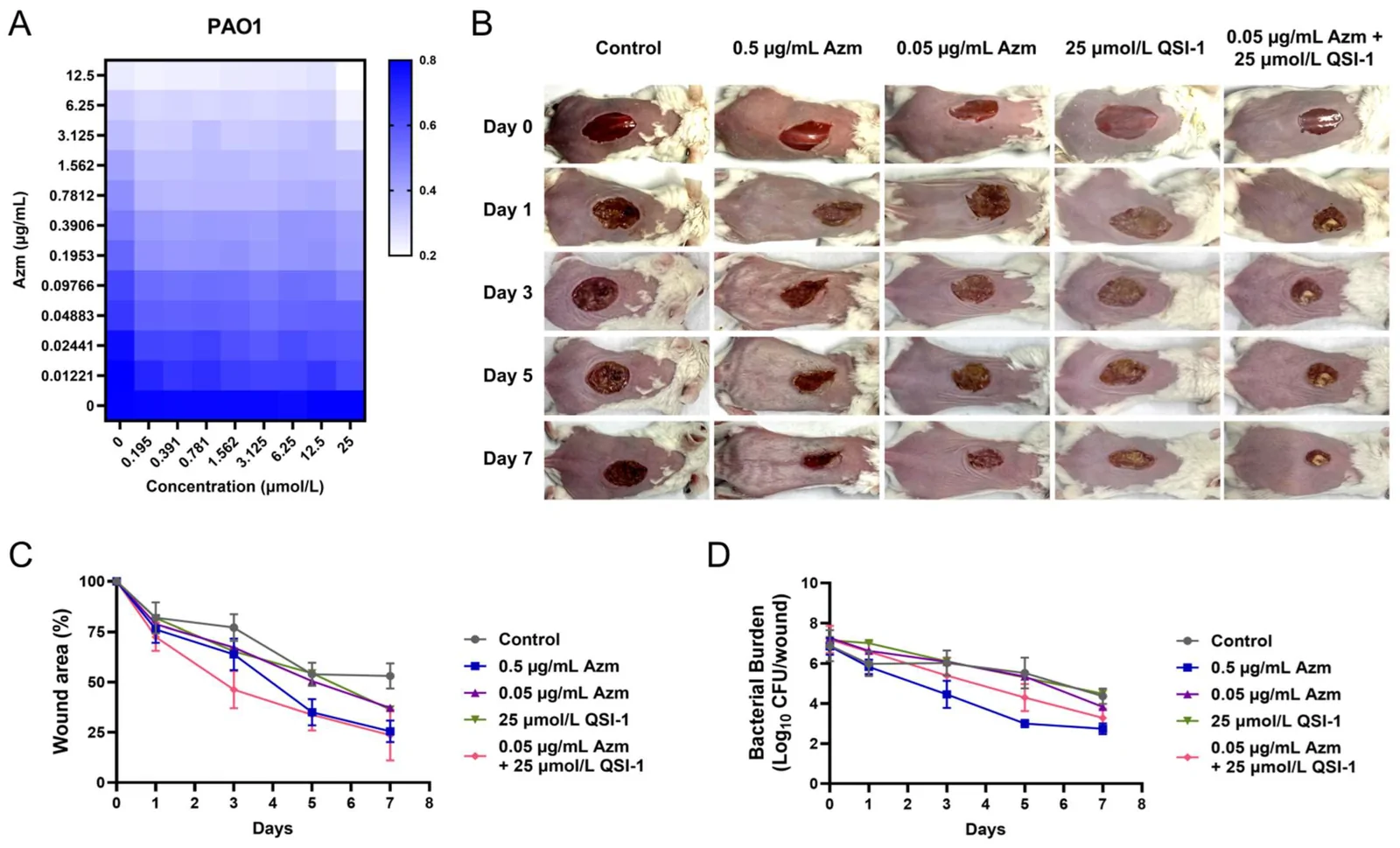
Azithromycin potentiation and in vivo efficacy of **QSI-1**. (**A**) Checkerboard broth microdilution of **QSI-1** (0–25 μmol/L) with azithromycin (Azm, 0–12.5 μg/mL) against *P. aeruginosa* PAO1, shown as OD_600_. (**B**) Representative wound images from days 0–7 for vehicle control, Azm (0.5 μg/mL), Azm (0.05 μg/mL), **QSI-1** (25 μmol/L), and Azm (0.05 μg/mL) + **QSI-1** (25 μmol/L). (**C**) Wound area expressed as a percentage of day 0. (**D**) Wound bacterial burden expressed as log_10_ CFU/wound over 7 days. Data are presented as mean ± SD.

**Figure 9:**
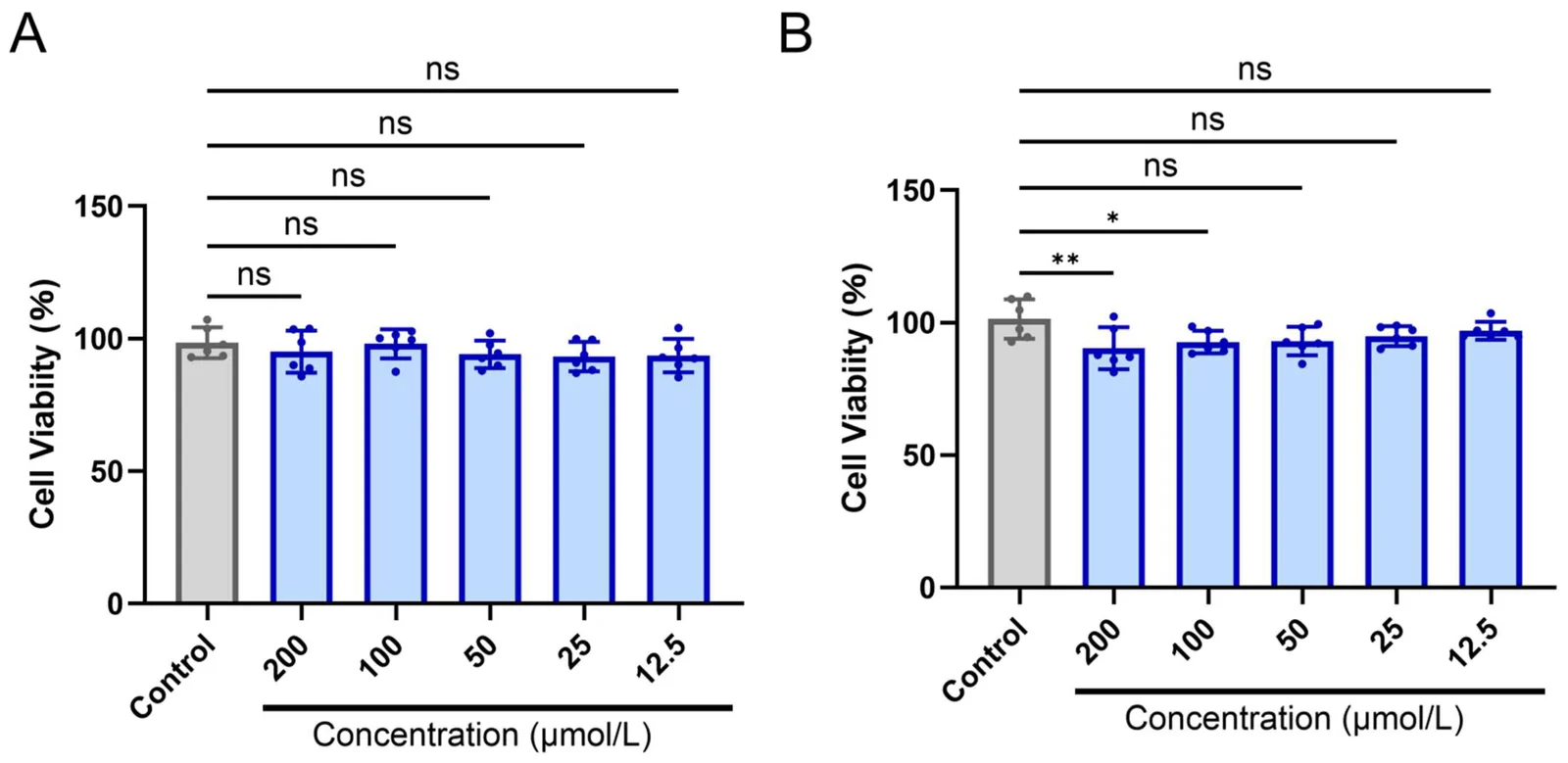
Cytotoxicity of **QSI-1**. Viability of (**A**) murine RAW264.7 macrophages and (**B**) human HEK293T cells after 24 h exposure to **QSI-1** (12.5–200 µmol/L) versus untreated control. * *p* < 0.05, ** *p* < 0.01; ns, not significant.

**Scheme 1:**
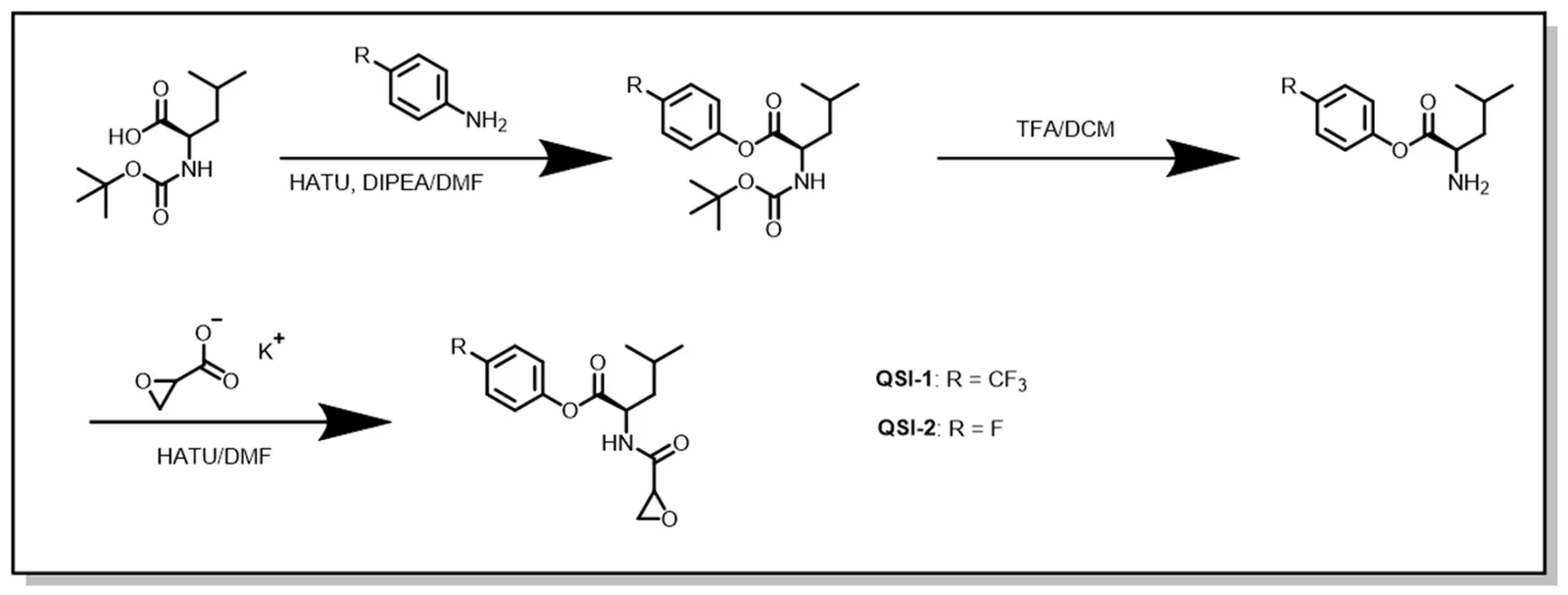
Synthesis of **QSI-1** and **QSI-2**. Reagents and conditions: (i) HATU, DIPEA, DMF, 0 °C to room temperature; (ii) TFA, DCM, room temperature; (iii) potassium oxirane-2-carboxylate, HATU, DIPEA, DMF, 0 °C to room temperature. R = CF_3_ for **QSI-1** and R = F for **QSI-2**.

## Data Availability

The data presented in this study are available on request from the corresponding author.

## Supplementary Materials

The following supporting information can be downloaded at: https://www.mdpi.com/article/10.3390/ijms27198465/s1.

## Abbreviations

The following abbreviations are used in this manuscript: ABTGC, ABT minimal medium supplemented with glucose and casamino acids; AMR, antimicrobial resistance; ANOVA, analysis of variance; CFU, colony-forming unit; CLSM, confocal laser-scanning microscopy; DCM, dichloromethane; DIPEA, N,N-diisopropylethylamine; DMF, N,N-dimethylformamide; DMSO, dimethyl sulfoxide; EPS, exopolysaccharide; ESI, electrospray ionisation; GFP, green fluorescent protein; HATU, 1-[bis(dimethylamino)methylene]-1H-1,2,3-triazolo [4,5-b]pyridinium 3-oxide hexafluorophosphate; HPLC, high-performance liquid chromatography; HRMS, high-resolution mass spectrometry; IC_50_, half-maximal inhibitory concentration; LB, lysogeny broth; LC-MS, liquid chromatography–mass spectrometry; MD, molecular dynamics; NMR, nuclear magnetic resonance; OD_600_, optical density at 600 nm; PBS, phosphate-buffered saline; PI, propidium iodide; QS, quorum sensing; RMSD, root-mean-square deviation; RMSF, root-mean-square fluctuation; SAR, structure–activity relationship; SD, standard deviation; TFA, trifluoroacetic acid; t-SNE, t-distributed stochastic neighbour embedding; TLC, thin-layer chromatography; WHO, World Health Organization.
