# Supplementary material for: Structure-Guided Covalent Design and Biological Evaluation of Glycidamido-Leucinamide Antivirulence Agents Against Pseudomonas aeruginosa
Source: Int J Mol Sci. 2026 Sep 23;27(19):8465. doi: 10.3390/ijms27198465 (PMC13665176; doi:10.3390/ijms27198465)
Supplement: Supplementary file 1 [file ijms-27-08465-s001.zip › ijms-4449910-supplementary.pdf]

Supporting Information

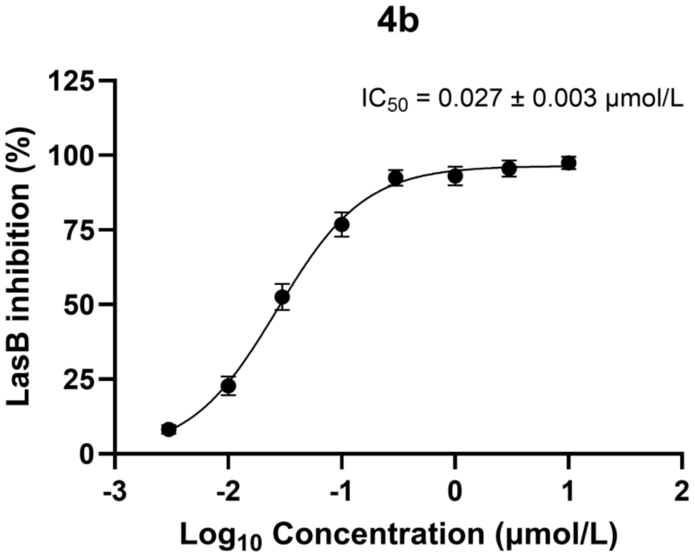

Figure S1. Inhibition of purified LasB by the reference inhibitor **4b**. Concentration-dependent inhibition of recombinant LasB by the structurally related phosphonate inhibitor **4b**. Data are presented as mean ± SD from three independent experiments.

^{1}H NMR spectrum of **QSI-1** in Chloroform-*d* (400 MHz).

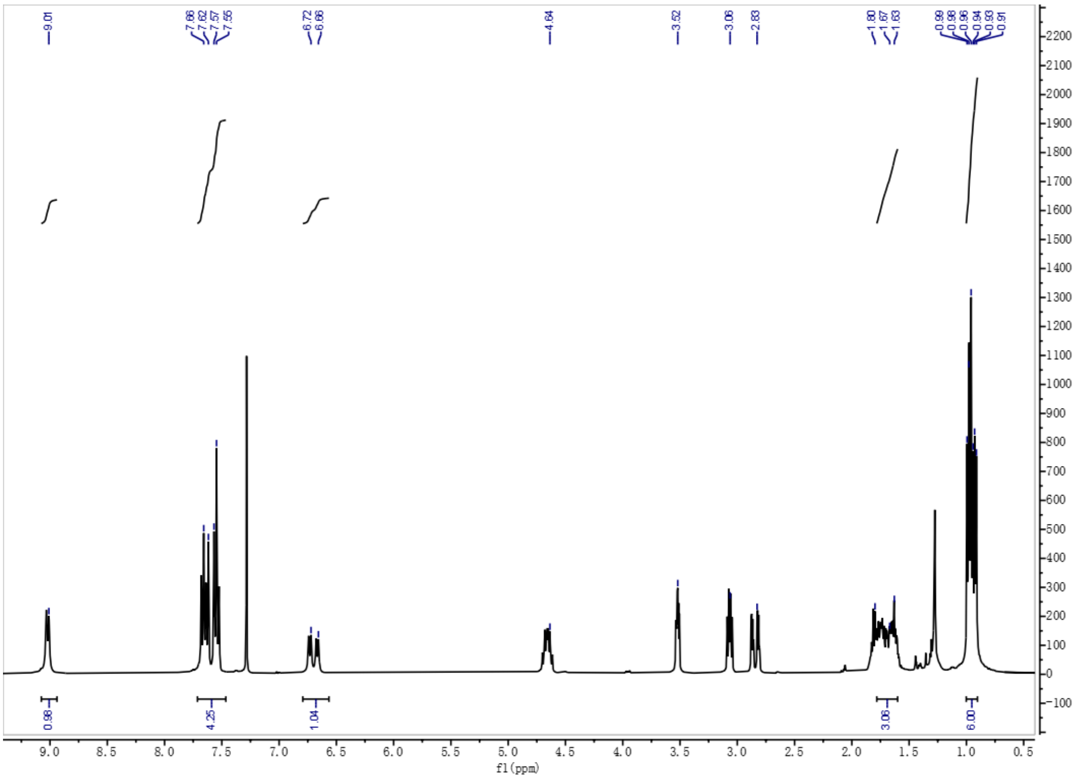

^{13}C NMR spectrum of **QSI-1** in Chloroform-*d* (101 MHz).

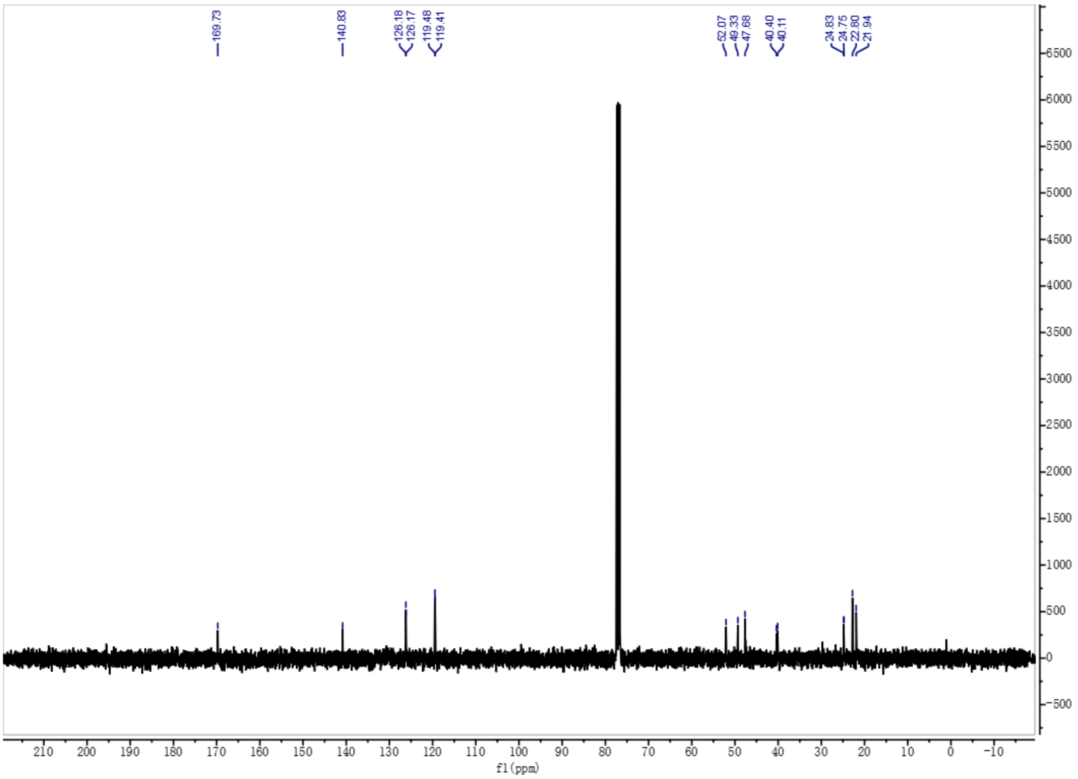

HRMS of **QSI-1**

$C_{16}H_{20}F_3N_2O_3$: +ESI Scan (rt: 8.73 min)

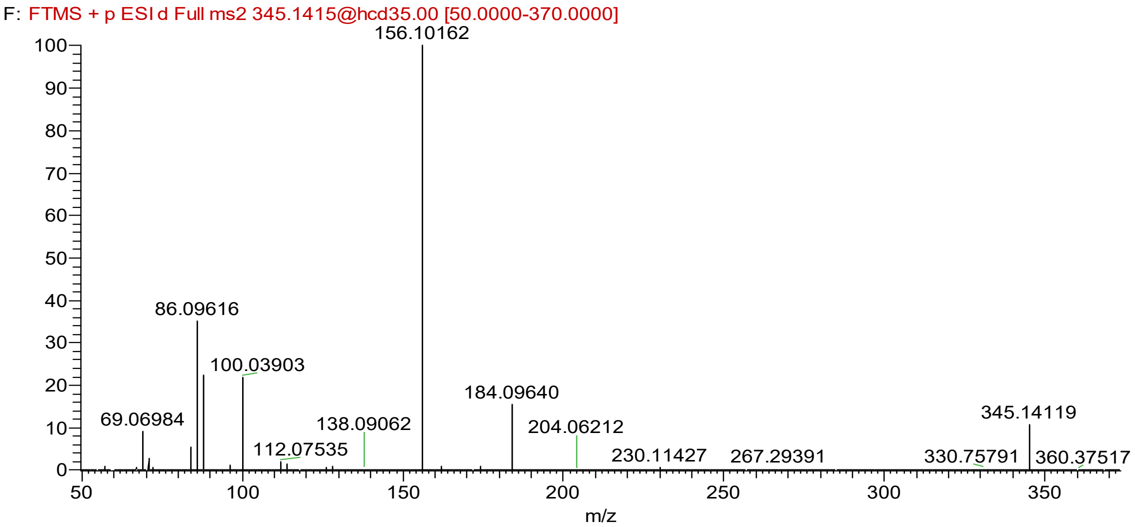

Counts *vs.* Mass-to-Charge (*m/z*)

^{1}H NMR spectrum of **QSI-2** in Chloroform-*d* (400 MHz).

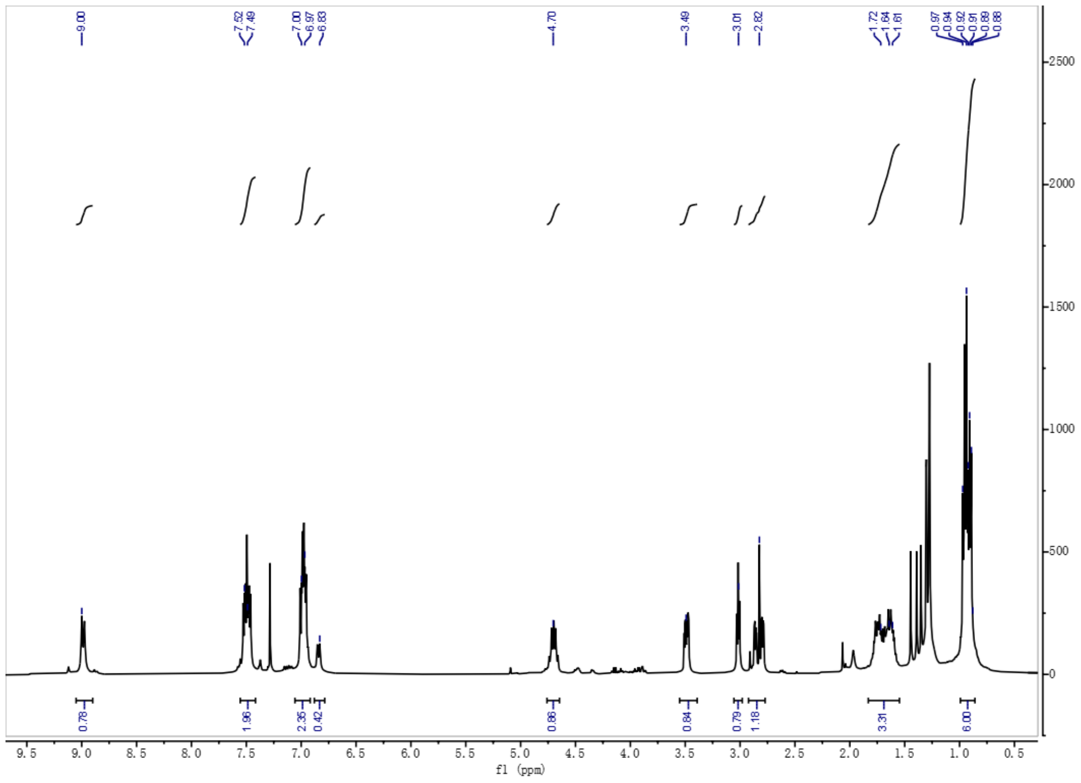

^{13}C NMR spectrum of **QSI-2** in Chloroform-*d* (101 MHz).

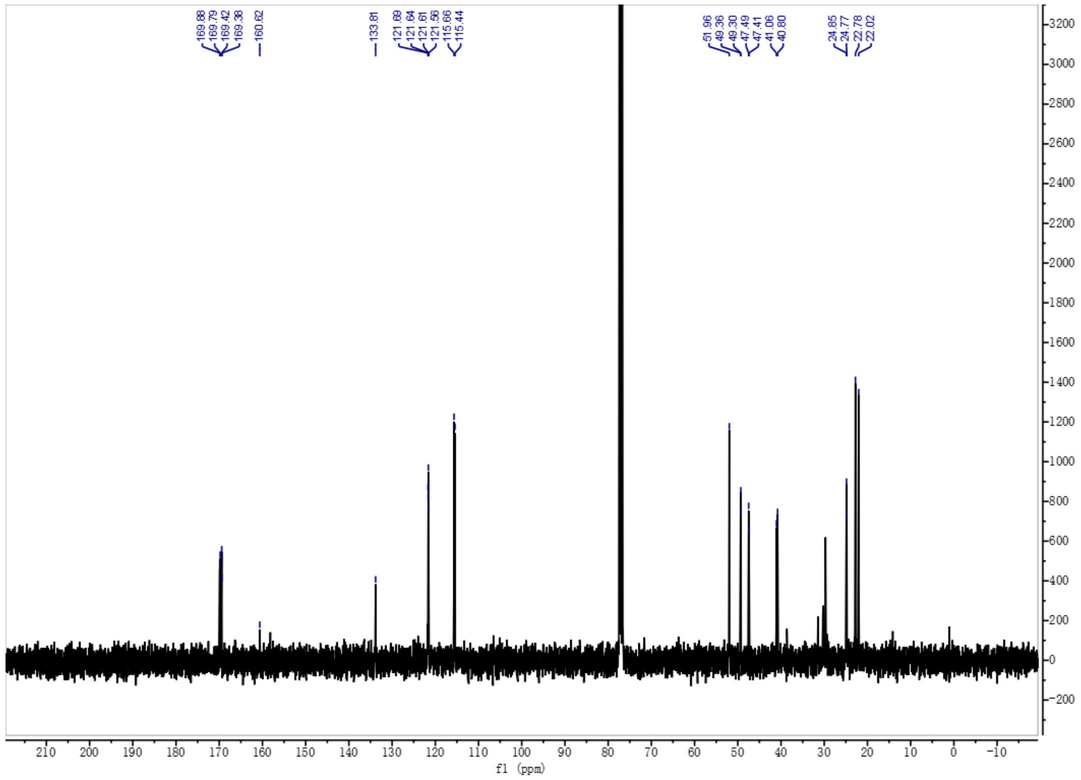

HRMS of **QSI-2**

$C_{15}H_{20}FN_2O_3$: +ESI Scan (rt: 8.73 min)

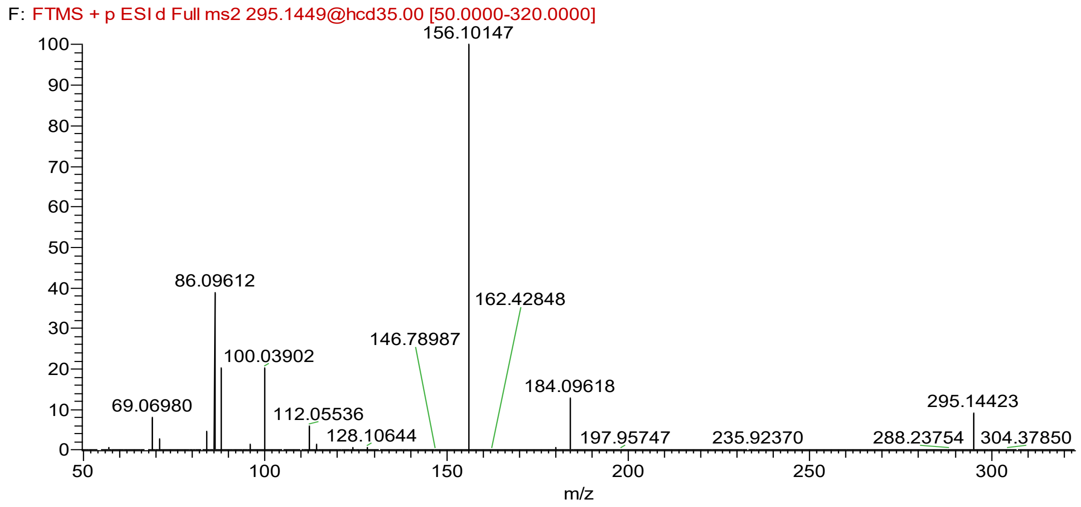

Counts *vs.* Mass-to-Charge (*m/z*)
